# Immunohistochemical Analysis of Postburn Scars following Treatment Using Dermal Substitutes

**DOI:** 10.1155/2022/3686863

**Published:** 2022-02-25

**Authors:** Mi Young Lee, Hyunchul Kim, In Suk Kwak, Youngchul Jang, Younghee Choi

**Affiliations:** ^1^Department of Physical Therapy, Sahmyook University, Hwarangro 815, Nowon-gu, Seoul 139-742, Republic of Korea; ^2^Department of Pathology, CHA Ilsan Medical Center, 1205 Jungang-ro, Ilsandong-gu, Goyang-si, Gyeonggi-do, Republic of Korea; ^3^Department of Anesthesiology and Pain Medicine, Hallym University Hangang Sacred Heart Hospital, Seoul, Republic of Korea; ^4^Department of Plastic Surgery, BPS Soo Hospital, 15F, Dosan-daero, Gangnam-gu, 06035 Seoul, Republic of Korea; ^5^Department of Pathology, Hallym University Dongtan Sacred Heart Hospital, Seoul, Republic of Korea; ^6^Research Institute for Complementary and Alternative Medicine, Hallym University, Republic of Korea

## Abstract

**Background:**

Post-burn hypertrophic scars commonly occur after burns. Studies that compare dermal substitutes with other treatment methods are insufficient. The purpose was to analyze the histopathological differences in hypertrophic burn scars after Matriderm®+split-thickness skin graft (STSG) and compare with AlloDerm®+STSG, STSG, full-thickness skin graft (FTSG), and normal skin.

**Methods:**

Samples of unburned, normal skin and deep 2^nd^ or 3^rd^ degree burns were obtained from patients who experienced a burn injury in the past to at least 6 months before biopsy, which was performed between 2011 and 2012. All subjects received >6 months of treatment before the biopsy. Intervention groups were normal (63), STSG (28), FTSG (6), Matriderm® (11), and AlloDerm® (18). Immunohistochemical analyses of elastin, collagen I, collagen III, cluster of differentiation 31 (CD31), smooth muscle actin (*α*-SMA), and laminin from scar and control tissues were performed and compared.

**Results:**

*α*-SMA vascular quantity and vessel width, stromal CD31, and basement membrane laminin expression were not significantly different between normal and intervention groups. Matriderm® group showed no significant difference in elastin, collagen III, stromal CD31 and *α*-SMA, CD31 vessel width, stromal *α*-SMA, vessel quantity and width, and laminin length compared to the normal group, meaning they were not significantly different from the normal skin traits.

**Conclusion:**

Dermal substitutes may be an optimal alternative to address the cosmetic and functional limitations posed by other treatment methods.

## 1. Introduction

The prevalence of hypertrophic scars after a burn injury is approximately 70% [1]. After a burn injury, it is common for one of the two types of pathologic scars to develop, such a hypertrophic scar or a keloid scar. Scars that are nonhypertrophic and unobtrusive are usually developed after superficial second-degree burns and are usually flat and pliable with slight discoloration [2]. However, pathologic scars such as hypertrophic scars develop with deeper burns. Postburn hypertrophic scars frequently occur in burn patients, which can affect cosmesis and cause adhesions and loss of function [3]. A hypertrophic scar does not grow beyond the boundaries of the original site of skin injury, is thick, red, and stiff, and may cause pain and itchiness [4, 5]. Previously, burn survival was mostly emphasized. However, patients often experienced limited ROM, scar contracture, dissatisfaction with the aesthetical results, and decreased quality of life [6]. A previous study surveyed 753 burn patients and found that 96% reported function, 85% reported pain and itching, and 59% reported cosmesis to be an important aspect of burn wound recovery. Cosmesis was particularly important to young, female patients with burns to the head and neck areas [7].

Nonoperative conservative treatment (CT) includes silicone, pressure garments, corticosteroid injections, and cryotherapy [8]. However, when compared to active surgical treatment, CT appears to be less effective in addressing issues related to elasticity, infectious complications, and pigmentation changes [9].

Split-thickness skin graft (STSG) involves excising the epidermis and part of the dermis where sufficient amount of reticular dermal tissue is left in the wound bed to enable self-skin regeneration [10]. Full-thickness skin graft (FTSG) consists of the epidermis, dermis, subcutaneous, and the epidermal appendages [11]. Grafting may lead to shorter average hospitalizations, reduce cost, healing time, cosmesis, and time away from work compared to CT [12, 13] but may not satisfy the issues regarding cosmesis and functionality [14]. FTSG is ideal for burn injuries due to less long-term contracture and scar formation but has limited donor sites, is reserved for small defects, may not address contour distortion, and may become hyperpigmented [14–16]. STSG is suitable for larger areas of injuries but can cause decreased cosmesis and increased risk of contracture [14, 16]. Therefore, recent studies have investigated dermal substitutes in its ability to produce favorable functional and aesthetic outcomes [17].

Dermal substitutes can be synthetic or biological materials. Synthetic skin substitutes are made of nonbiological molecules and polymers that are not present in the normal skin [18]. Natural biological materials contain human cadaveric (gold standard) or porcine tissue, which is treated to obtain an acellular scaffold [18].

AlloDerm® (LifeCell Corporation, Woodlands, TX) contains human cadaver tissue and is one of the oldest and most utilized matrices [19]. AlloDerm® can serve as an acellular template containing natural dermal pores for regeneration, is immunologically inert [20], has an intact basement membrane [14], and can be applied with a one-stage process where it is applied followed by the application of a thin STSG [21]. Risk of transmitting infectious diseases, high cost, not being as readily available, and having a relatively shorter shelf-life (2-3 years) are some disadvantages [22, 23]. Previous studies have examined the use of AlloDerm®+STSG compared with STSG alone and have shown improved elasticity and pliability [18], cosmetic concerns [21], and overall scar quality and functionality [20, 24, 25].

Matriderm® (Dr. Otto Suwelack Skin & Health Care AG, Billerbeck, Germany) is a multiporous membrane from bovine origin and contains collagen types I, III, and V and hydrolysate of elastin alpha [26]. It was developed to improve the healing process of full-thickness wounds and is rendered to be effective in decreasing contractures and enhancing skin elasticity and thus improving function and aesthesia [27]. Matriderm® is relatively easy to apply and is cost-effective and less time-consuming in that it can be performed in a one-stage procedure with STSG when using a 1 mm thick matrix [27, 28]. Xenografts, such as Matriderm®, generally have a shelf-life of 18 months to 5 years [14]. Studies support the use of Matriderm®+STSG compared with STSG alone and have found significantly improved pliability [27] and less pronounced and homogenous collagen deposition [28].

Studies have investigated the treatment effects, such as take rates, vascularization, pliability, epidermal water loss, scar thickness, neodermal thickness, collagen deposition, and range of motion (ROM) [17, 27, 28]. Histological analysis has been performed for elastic and collagen fibers, epidermal thickness, myofibroblasts, fibroblasts, and mast cells [29–31]. However, previous studies have investigated only one or two types of treatment techniques, such as graft (STSG and FTSG) vs CT, STSG vs FTSG, Matriderm® vs CT, Matriderm® vs STSG, AlloDerm® vs CT, and Matriderm® vs Integra® [13, 17, 21, 32, 33].

Studies that compare various treatment methods for postburn hypertrophic scars in humans on histopathological characteristics that indicate satisfactory healing outcomes are insufficient. Therefore, the purpose of this study was to analyze the histopathological differences in elastin, collagen I, collagen III, vascularity, cluster of differentiation 31 (CD31), laminin, and smooth muscle actin (*α*-SMA) expression levels in hypertrophic burn scars and compare with after the application of Matriderm®+STSG, AlloDerm®+STSG, STSG, and FTSG as well as with normal, unburned skin of human subjects. It is hypothesized that the use of dermal substitutes will yield increased elastin levels, optimal levels of collagen so that it is not excessive, increased CD31 and decreased *α*-SMA levels, and increased laminin intensities in scar tissue so that it is comparable to the normal skin.

## 2. Materials and Methods

### 2.1. Patient Selection

All tissue biopsies were obtained during reconstructive or cosmetic surgery from patients who experienced a burn injury in the past years to least 6 months before the biopsy (Table 1). All patients had received treatment for at least 6 months before the biopsy. Normal, unburned skin samples were obtained approximately 5-10 cm away from the wound edge. Normal and hypertrophic scar tissue samples were obtained from the same patient. A total of 89 patients from 2011 to 2012 from the Hallym University Hangang Sacred Heart Hospital in Korea were included. Subjects were included if they experienced a deep second- or third-degree burn and had developed a hypertrophic scar and were excluded if they (i) were pregnant, (ii) were less than 8 years old, (iii) had any presence of psychotic diseases (iv) have cancer and active infection, or (v) had prior treatments with immunomodulators, ultraviolet (UV) irradiation, or hydrogen peroxide 3 months prior to surgery. Of the 89 subjects, 3 were excluded due to not meeting the inclusion or exclusion criteria. Of the 86 subjects, 23 were excluded for the missing data of the independent variables. The remaining 63 subjects were divided into five groups according to the postburn hypertrophic scar treatment received for at least 6 months (control = 63, STSG = 28, FTSG = 6, Matriderm® = 11, and AlloDerm® = 18).

### 2.2. Informed Consent

Prior to surgery, all patients had provided their informed consent for research purposes. This study was approved by the Institutional Review Board (IRB) of the Hallym University Hangang Sacred Heart Hospital (IRB approval No. 2012-180).

### 2.3. Histopathological Analyses and Data Collection

All tissue samples were obtained using punch biopsy (6 mm) during surgery from burn injury sites of patients between 2011 and 2012. All burns were classified as 3^rd^-degree or deep 2^nd^-degree burns. Control tissue samples were obtained from unburned areas approximately 5-10 cm away the burn injury site. After placing the tissue samples in 10% neutral buffered formalin for 18 hours, they were subsequently processed with Paraplast (Sigma-Aldrich, St. Louis, MO, USA) for paraffin embedding. For routine histology purposes, serial 5 *μ*m thick tissue sections had been processed. Immunohistochemical staining was performed by using the Benchmark Ultra Autoimmunostainer (Ventana Medical Systems, Inc., USA) and the Optiview DAB IHC Detection Kit (Ventana Medical Systems, Tucson, AZ, USA). The following antibodies were used: rabbit polyclonal anticollagen III for collagen III (NB100-92162, 200x dilution, enzyme treatment, Novus Biologicals, Littleton, CO, USA), mouse monoclonal antibody for *α*-SMA (NB600-536, 400x dilution), NB600-408 for collagen I (200x dilution), NBP1-80710 for elastin (200x dilution), NB600-562 for CD31/PECAM (200x dilution, and NBP1-50430 for laminin (Novus Biologicals, Littleton, CO, USA). All dependent variables were assessed from areas that were not further down past the reticular dermal layer.

### 2.4. Histopathological Analyses of Elastin, Collagen III, and Collagen I

For elastin and type I and type III collagen fibers, a positive reaction was observed as brown staining of fibers. The area of the positive immunostaining was viewed using a Nikon microscope (Plan-Apo, Nikon, Tokyo, Japan) and was quantified using a digital camera (Nikon, DS-Ri3, Nikon digital SLR camera FX-format CMOS sensor optimized for microscopy, Tokyo, Japan) and an image analyzer program (NIS-Elements BR, version 5.01, 64 bit, Nikon, Tokyo, Japan). Original images of the fibers at 200x magnification for elastin, 100x for collagen III, and 200x for collagen I were converted into red, green, and blue (RGB) images where the fibers were in maximal separation from background tissues [33]. A rectangular region of interest (ROI) of 154.9 in width and 179.1 in height measured in pixels was used to calculate the ratio of positive to negative staining from three random areas (*μ*m^2^). Mean values of the three randomly selected fields of view (areas (*μ*m^2^)) within the ROI were obtained [34].

### 2.5. Histopathological Analyses of *α*-SMA and CD31

*α*-SMA and CD31 cells in the stroma were counted within a built-in 10 × 10 grid from the three different areas with a digital camera. Each section of the grid measured 25 *μ*m at 400x magnification [35]. Mean values were obtained from the three measurements. Vessels expressing *α*-SMA and CD31 were quantified based on ten separate areas within a 10 × 10 grid at a magnification of 400x and were averaged. Vessel length and width was measured in millimeters.

### 2.6. Histopathological Analyses of Laminin

The intensity of laminin expression in the basement membrane was quantified and averaged using a scale of 1 to 4, where 4 represented strong intensity at a magnification of 40x, 3 at 100x, 2 at 200x, and 1 at 400x [36]. The greater the intensity, the lesser the magnification level required for observation. The laminin length among the basement membrane was also quantified and averaged based on a scale of 1-4, where 4 is equal to laminin being observed among the full length of the tissue sample (100%), 3 at 75%, 2 at 50%, and 1 at 25% or less at 100x magnification.

The assessor was blinded from the interventions when assessing the variables from the tissue sample slides.

### 2.7. Statistical Analyses

Statistical analyses were performed using SPSS version 21.0 for Windows (SPSS Korea, Inc., Seoul, Korea). The Kruskal-Wallis was used to compare for the laminin variables between groups. The one-way ANOVA was used to make comparisons of the treatment effect between the groups for all other variables. The Bonferroni method was used for post hoc analysis. Subjects with missing independent variables were excluded from the analysis. Significance levels were set at *p* < 0.05.

## 3. Results

**Figure 1:**
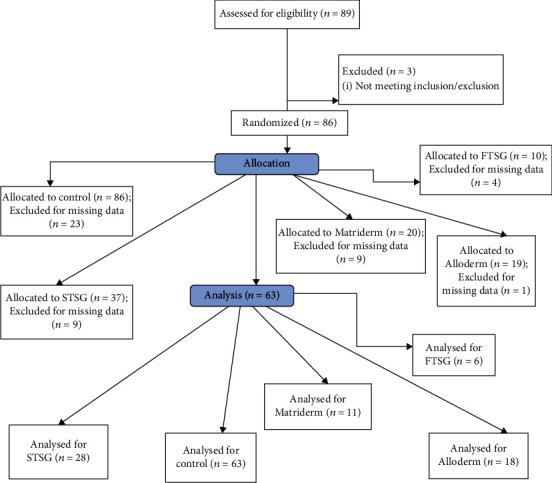
Study flow diagram. Details of the study selection process.

**Figure 2:**
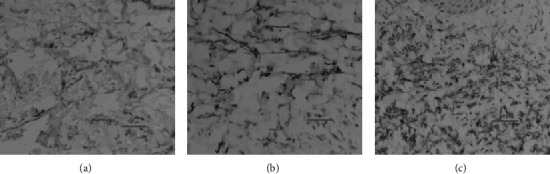
Comparison of elastin fibers. Comparison of elastin fibers of the normal (a), STSG (b), and AlloDerm® (c) groups. 200x magnification, 100 *μ*m scale bar; immunostaining technique. Elastic fibers are responsible for resilience and recoil in many tissues. There was a significant decrease in elastin fibers in the STSG and AlloDerm® groups compared to the normal group. Therefore, scar tissues in these two groups may be stiffer compared to the other groups.

**Figure 3:**
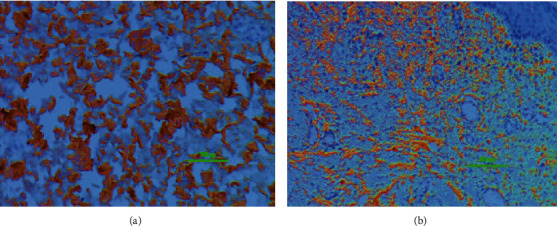
Comparison of collagen III fibers. Collagen III fibers of the normal (a) and STSG (b) group; 200x magnification, 100 *μ*m scale bar; immunostaining technique; Collagen provides tensile strength but hypertrophic scars contain an overabundance of collagen, contributing to a raised appearance and stiffness. There was a significant decrease in collagen III in the STSG group compared to the normal group.

Tissue samples from sixty-three subjects were categorized into five groups based on the intervention received for over 6 months (normal = 63, STSG = 28, FTSG = 6, Matriderm® = 11, and AlloDerm® = 18). The study selection process is shown in Figure 1. The general burn characteristics of the subjects are presented in Table 1. The histopathological characteristics of the intervention groups are presented in Table 2. This study examined and compared the presence of elastin, collagen, CD31, *α*-SMA, and laminin levels in both postburn hypertrophic scars and adjacent, unburned areas. There was no significant difference in CD31 in the stroma, *α*-SMA vascular quantity, *α*-SMA vessel width, and laminin expression in the basement membrane all groups, indicating that values were not significantly different between the normal and the intervention groups (Table 2). Significant differences in mean elastin were observed, particularly between STSG and AlloDerm® with the normal group (352.63 and 393.34 vs 754.24 *μ*m^2^, respectively; *p* < 0.05) (Figure 2). Collagen III amounts were significantly different, particularly between STSG and the normal group (2475.38 vs 4140.63 *μ*m^2^, respectively; *p* < 0.05) (Figure 3). There was a significant difference in collagen I, especially between the STSG, Matriderm®, and AlloDerm® groups compared to the normal group (2383.47, 2251.31, and 2350.55 *μ*m^2^ vs 3200.63 *μ*m^2^, respectively) and FTSG compared with STSG, Matriderm®, and AlloDerm® groups (4695.31 vs 2383.47, 2251.30, and 2350.55 *μ*m^2^, respectively; *p* < 0.05) (Table 2).

**Figure 4:**
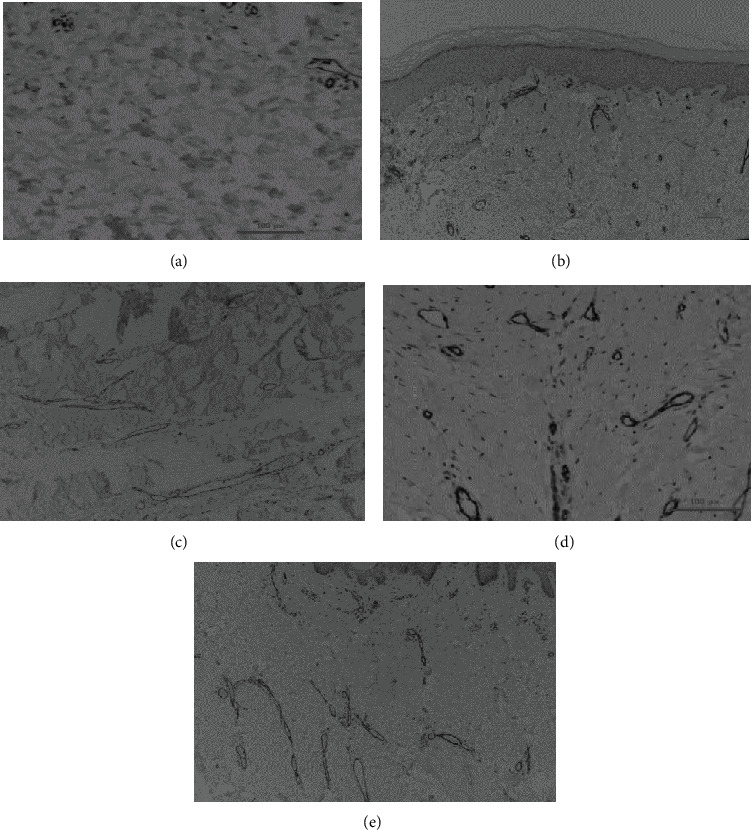
Comparison of CD31 vessel quantity. CD31 vessel quantity in the normal (a), STSG (b), FTSG (c), Matriderm® (d), and AlloDerm® (e) groups, respectively; 100x magnification, 100 *μ*m scale bar; immunostaining technique; Hyperactive fibroblasts in hypertrophic scars secrete higher than normal levels of angiogenic factors, which promote endothelial cell proliferation and more microvessel formation. CD31 helps to promote vascular barrier function in response to inflammatory stimuli. There was a significant increase in vessel quantity expressing CD31 in the FTSG and Matriderm® groups compared to the normal group.

There was a significant difference in CD31 vessel numbers, particularly between the FTSG and Matriderm® groups with the normal group (6 and 6 vs 4, respectively; *p* < 0.05) (Figure 4 and Table 2).

**Figure 5:**
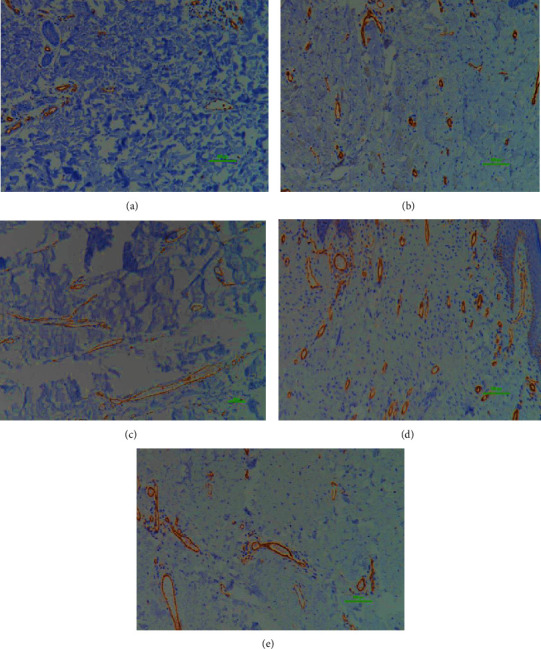
Comparison of CD31 vessel length. CD 31 vessel length of the normal (a), STSG (b), FTSG (c), Matriderm® (d), and AlloDerm® (e) groups; 100x magnification, 100 *μ*m scale bar; immunostaining technique; local collagen overproduction by hypertrophic scar fibroblasts mechanically squeezes the microvessels, thus leading to narrowing and deformation. CD31 helps to promote vascular barrier function in response to inflammatory stimuli. There was a significant increase in CD31 impregnated vessel length in the STSG, Matriderm®, and AlloDerm® groups compared to the normal group.

There was a significant difference in CD31 vessel length, especially between the Matriderm® and AlloDerm® groups with the normal group and STSG groups (32 and 32 vs 16 and 18 *μ*m, respectively; *p* < 0.05) (Table 2 and Figure 5).

**Figure 6:**
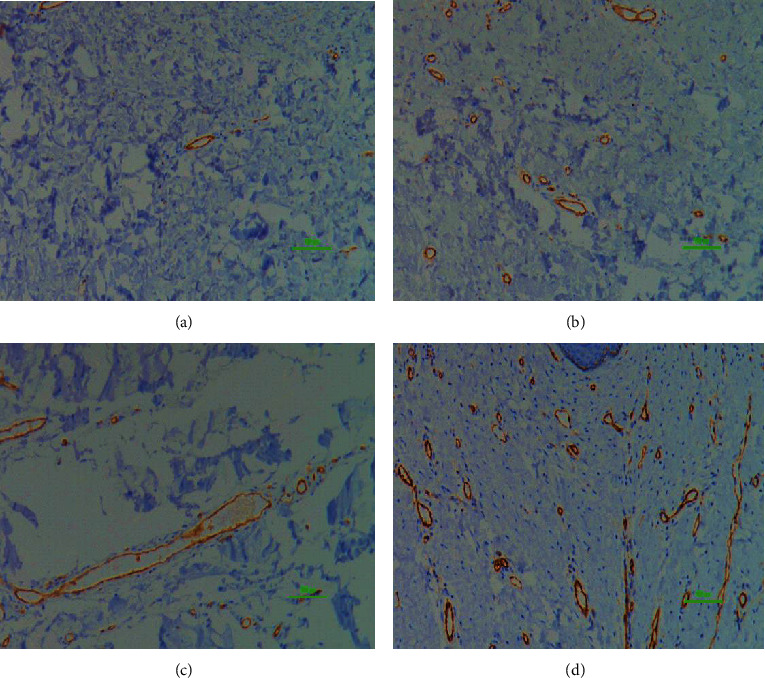
Comparison of CD31 vessel width. Comparison between control (a), STSG (b), FTSG (c), and Matriderm® (d); 100x magnification, 100 *μ*m scale bar; immunostaining technique; local collagen overproduction by hypertrophic scar fibroblasts mechanically squeezes the microvessels, thus leading to narrowing and deformation. CD31 helps to promote vascular barrier function in response to inflammatory stimuli. There was a significant increase in CD31 impregnated vessel width in the FTSG compared to the control, STSG, and Matriderm® groups.

There was a significant difference in CD31 vessel width, particularly between the FTSG with the normal, STSG, and Matriderm® groups (50 vs 20, 16, and 18 *μ*m, respectively; *p* < 0.05) (Table 2 and Figure 6).

**Figure 7:**
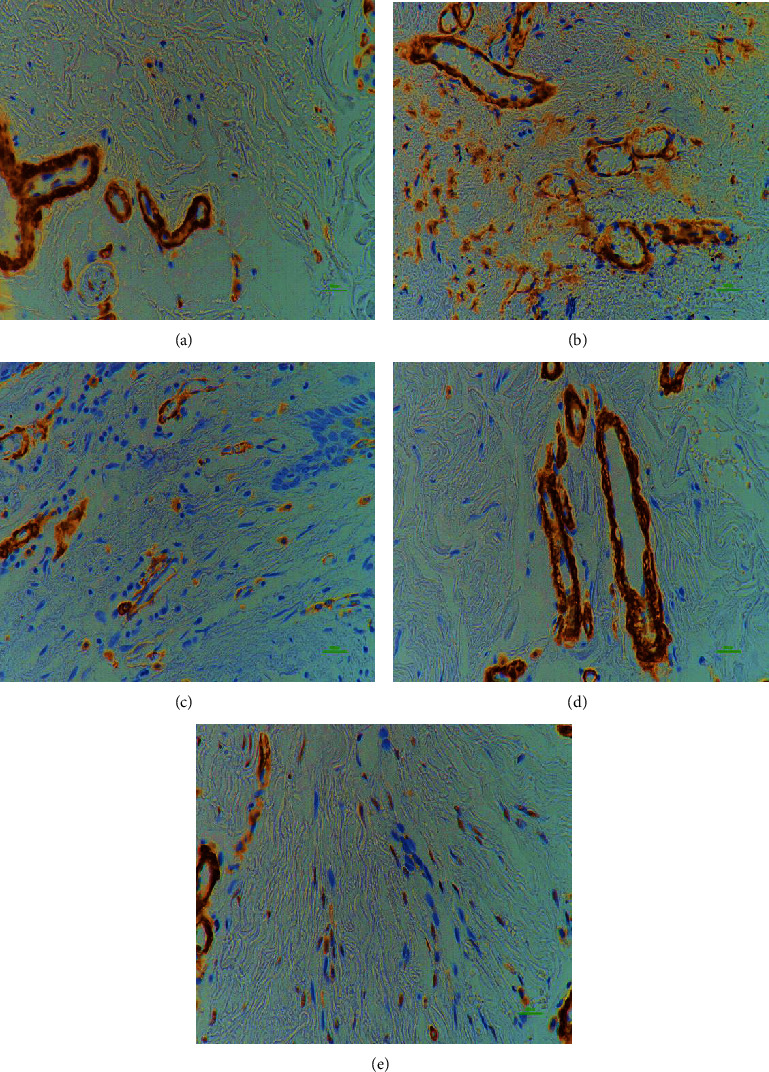
Comparison of *α*-SMA stroma. *α*-SMA of the normal (a), STSG (b), FTSG (c), Matriderm® (d), and AlloDerm® (e) group; 400x magnification, 100 *μ*m scale bar; immunostaining technique; Myofibroblasts express *α*-SMA, and persistence of myofibroblasts may lead to excess scarring, which impairs function and aesthetics. There was a significant increase in *α*-SMA in the STSG, FTSG, and AlloDerm® compared with the normal and Matriderm® groups.

There was a significant difference in *α*-SMA stroma between the STSG, FTSG, and AlloDerm® groups with the normal group (23, 25, and 33 vs 7, respectively; *p* < 0.05) and between the Matriderm® and AlloDerm® groups (10 vs 33, respectively; *p* < 0.05) (Table 2 and Figure 7).

**Figure 8:**
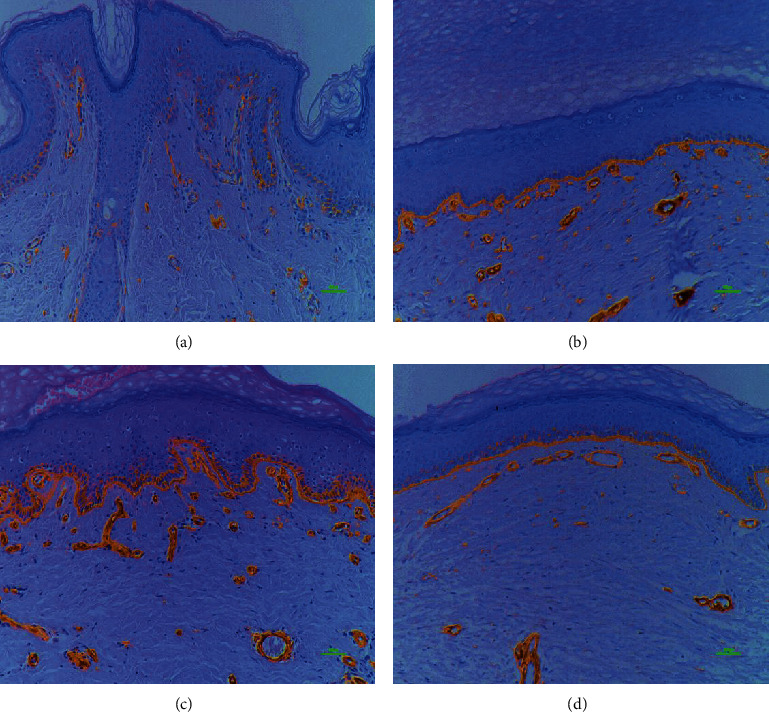
Comparison of laminin basement membrane intensity. Intensity of laminin in the basement membrane of the normal (a), STSG (b), Matriderm® (c), and AlloDerm® groups (d); 400x magnification, 100 *μ*m scale bar; immunostaining technique; laminins have a central role in formation, the architecture, and the stability of basement membranes, thus making them essential for basement membrane assembly. There was a significant increase in laminin expression intensity in the STSG, Matriderm®, and AlloDerm® groups compared with the normal group.

There was a significant difference in laminin intensity in the basement membrane, especially between the STSG, Matriderm®, and AlloDerm® groups with the normal group (3.22, 3.20, and 2.75 vs 2.56, respectively; *p* < 0.05) (Table 2 and Figure 8).

## 4. Discussion

### 4.1. Elastin

Elastic fibers are ECM components responsible for tissue resilience and recoil and are important properties for the skin, lungs, and vessels [37]. Devastating conditions may occur due to defects or abnormalities in elastic genes. Elastin proteins are important for cell signaling and induce many pathways including fibroblast migration and proliferation, keratinocyte migration, smooth muscle proliferation, ECM production and degradation, and cell survival [37]. Elastin-based dermal substitutes may improve scar function and appearance, reduce wound contraction, and assist in wound healing through combining the mechanical and cell signaling properties of elastin [38]. In this study, elastin fibers were significantly decreased in the STSG and AlloDerm® groups compared with the normal skin (*p* < 0.05) (Table 1). In contrast, elastic fibers in the FTSG and Matriderm® were not significantly different from the normal skin, indicating that the elasticity of the area with Matriderm® and FTSG was similar to the normal skin (Table 1). Although AlloDerm® also contains elastin, we suggest that its effects may not have been manifested as profoundly as Matriderm® and FTSG.

### 4.2. Collagen

Increased collagen types I and III levels are exhibited in hypertrophic scars compared with other collagen subtypes [39]. With scar tissue, type I collagen fibers are thick and morphological differences between normal and scar tissue occur probably due to the progressive accumulation of type I collagen [34]. Decreased collagen I and III levels in STSG, Matriderm®, and AlloDerm® groups compared to the normal skin may indicate that these intervention methods were able to address the prevention of a collagen overabundance. However, the mechanism involved in collagen reduction is not precisely known. Although it was not significantly greater than in the normal skin, collagen I and III levels were the highest in the FTSG group compared to the other intervention groups (Figure 4). The reason for this is not clearly known, but Yang et al. [40] found in their study that even at 6 months posttransplantation, 54 out of 60 nude mice that received full-thickness human skin graft transplantation developed hypertrophic scars. Increased collagen deposition and inflammatory infiltration were shown in these scars based on histologic exams. In contrast, no hypertrophic scars were developed in mice that received the full-thickness rat skin graft transplantation.

### 4.3. Cluster of Differentiation (CD31)

DeLisser et al. stated that PECAM-1/CD31 is involved in angiogenesis and suggests that the endothelial cell-cell adhesion molecule interactions are essential in creating new vessels, which are essential for supplying oxygen and nutrients to augment the rapid growth of cells that mediate repair [24, 41]. In hypertrophic scars, microvessel formation and collagen production are promoted by increased angiogenic and fibrogenic factors [11].

Local collagen overproduction by hypertrophic scar fibroblasts mechanically squeezes the microvessels, thus leading to narrowing and deformation [11]. CD31 is expressed on endothelial cells and serves as a cell adhesion and signaling receptor [42]. CD31 can promote barrier function through Rap1 and has an important role in restoring endothelial cell barrier function through rapid endothelial cell-cell junction assembly [43]. Studies in C57BL/6 mice showed that CD31 can prominently serve to dampen the inflammatory response in various acute and chronic inflammatory conditions, such as collagen-induced arthritis [43, 44]. Therefore, the presence of increased CD31 enhances vascular barrier function and can mitigate the inflammatory response.

It is reported in the literature that HTS regression leads to endothelial dysfunction [45], which ultimately means decreased CD31. It is possible that the majority of the scars in the Matriderm and the FTSG group were “younger” compared to the scars in the other groups, which may explain the increased CD31 levels found in the Matriderm and FTSG group.

### 4.4. Smooth Muscle Actin (*α*-SMA)

Hypertrophic scars contain abundant nodules containing myofibroblasts, which contain *α*-SMA [46] Myofibroblasts are differentiated fibroblasts found in granulation tissue and fibrotic lesions [28, 29, 47] and are responsible for skin contraction after wounding [46]. A large proportion of myofibroblasts express *α*-SMA, and persistence of myofibroblasts may lead to excess scarring, which impairs function and aesthetics [35, 48]. Significant increases in *α*-SMA stroma expression levels in the STSG, FTSG, and AlloDerm® groups were observed compared to the normal skin (*p* < 0.05). There was also a significant increase in *α*-SMA stroma levels in the AlloDerm® compared to the Matriderm® group (*p* < 0.05). van den Broek et al. stated that with hypertrophic scars, *α*-SMA positive staining was present not just around blood vessels but in single cells in the lower dermis as well [46]. In our study, there was no significant difference in *α*-SMA in the stroma between the Matriderm® group and in the normal skin in which both had low cell numbers compared to the other intervention groups (*p* > 0.05). In normotrophic scar and normal skin, *α*-SMA staining is mainly restricted to the blood vessels [46]. Failure of appropriate downregulation of wound healing cells or the extended presence of pathological wound healing signals may occur due to the upregulation of fibroblast contractile activity by excess *α*-SMA levels [48, 49]. Thus, lower *α*-SMA levels may indicate a decrease in fibroblast contraction [48], which can reduce the development of scar contraction [50].

### 4.5. Laminin

Laminins have a central role in the formation, architecture, and stability of basement membranes [51] and are particularly needed for the assembly of the ECM. Laminins are heterodimers constituted by the association of *α*, *β*, and *γ* chain gene products [52]. Our study showed that the length of laminin expression in the basement membrane was not significantly different in the intervention groups compared with the control group (*p* > 0.05). However, laminin expression intensity in the basement membrane was significantly increased in the STSG, Matriderm®, and AlloDerm® groups compared with the normal skin (*p* < 0.05). According to Xie et al., increased laminin expression may be due to HOXB9 mechanisms. Co-IP assays have shown that HOXB9 directly interacts with ERK, JNK, and p38. This interaction may cause p-p38, p-ERK, and p-JNK to accumulate, which activates MAPK and subsequently increases laminin, FN, and Col1 expression levels, which ultimately results in ECM reconstruction since it has been damaged due to burn injury [53]. According to this study, STSG, Matriderm®, and AlloDerm® appear to be effective in restoring laminin in the basement membrane postburn injury. There was no significant difference in elastin, collagen, or *α*-SMA, which are essential factors that contribute to scar pliability and appearance, when compared with the normal skin. Hur et al. also reported that application of artificial dermis that consists of elastin is effective functionally and aesthetically by decreasing contractures and enhancing skin elasticity in humans [54]. In addition, for damaged skin, restoring the protective functions of the skin in a timely manner is key to successful treatment [55].

### 4.6. Limitations

There were some limitations of this study. The sample size was not uniform in all the intervention groups. The exact total body surface area (TBSA) was not known, and there was no pre-postintervention assessment or comparisons made. In addition, functional outcomes such as skin elasticity, relief, thickness, pliability, and itch or the Patient and Observer Scar Assessment Scale or the Vancouver Scar Scale outcome measures were not assessed. However, this study has given light to the histopathological differences in postburn hypertrophic scars after the application of various interventions that are commonly reported in the literature. Further research should include a more uniform sample size as well as a pre-postintervention assessment of other functional outcomes to make further comparisons.

In conclusion, although autografting is a common burn intervention and is preferable compared to CT, it does not satisfy the issues regarding cosmesis and functionality. Therefore, artificial dermal substitutes may be considered as an optimal alternative to address these limitations. Of all the treatment approaches, the most favorable results in elastin, collagen, CD31, *α*-SMA, and laminin appeared in the group treated with Matriderm®, which are the variables important for healing, function, and cosmesis. Although further studies are warranted to confirm these findings, this study suggests that Matriderm® may be considered as an important component for treatment of burn wounds.

## Figures and Tables

**Table 1:** General burn characteristics of subjects according to interventions (*n* = 63).

Variable	STSG (28)	FTSG (6)	Matriderm® (11)	AlloDerm® (18)	*p*
Age (yrs)	27.32 ± 12.12	42.00 ± 12.72	44.60 ± 15.31	26.44 ± 11.17	0.051
Side (R : L)	12 : 16	2 : 4	6 : 5	9 : 9	
Burn site	LE (8), trunk (6), UE (10), and face/neck (4)	UE (4) and face/neck (2)	LE (2), trunk (1), UE (5), and face/neck (3)	LE (6), inguinal (1), and UE (11),	
Burn source	Flame (11), hot water (6), electric (4), iron (2), friction (2), steam (2), and chemical (1)	Flame (4), hot water (1), and electric (1)	Flame (7), electric (2), iron (1), and friction (1)	Flame (8), hot water (5), electric (1), iron (1), steam (2), and contact (1)	
Gender (M : F)	22 : 6	5 : 1	9 : 2	11 : 7	
Duration between graft and biopsy (months)	15.21 ± 5.73	16.50 ± 5.61	13.50 ± 4.83	16.00 ± 6.54	0.481
Duration between burn and biopsy (months)	19.74 ± 10.40	83.60 ± 120.18	21.73 ± 17.36	30.25 ± 24.14	0.079

Mean ± standard deviation. STSG: split-thickness skin graft; FTSG: full-thickness skin graft; LE: lower extremity; UE: upper extremity *p* < 0.05 considered significant.

**Table 2:** Histopathological comparison of normal and scar tissue characteristics according to intervention (*n* = 63).

Variable	Normal (63)	STSG (28)	FTSG (6)	Matriderm® (11)	AlloDerm® (18)	*F*	*p*
Elastin (*μ*m^2^)	754.24 ± 519.4	352.63 ± 321.97^∗^	779.98 ± 627.66	409.18 ± 288.67	393.34 ± 283.79^∗^	3.238	0.014
Collagen III (*μ*m^2^)	4140.63 ± 2892.21	2475.38 ± 1602.65^∗^	3633.88 ± 2656.24	2984.91 ± 1331.42	2743.35 ± 1380.35	2.722	0.033
Collagen I (*μ*m^2^)	3200.63 ± 1778.89	2383.47 ± 746.76^∗^	4695.31 ± 1303.40°	2251.30 ± 1210.39^∗^^∞^	2350.55 ± 1418.80^∗^^∞^	3.116	0.008
CD31 stroma (*n*)	6 ± 4	8 ± 3	5 ± 1	4 ± 1	6 ± 4	2.285	0.066
CD31 vessel (*n*)	4 ± 1	5 ± 2	6 ± 2^∗^	6 ± 3^∗^	5 ± 2	4.508	0.002
CD31 length (*μ*m)	16 ± 10	18 ± 11	22 ± 6	32 ± 24^∗^°	32 ± 16^∗^°	5.435	0.001
CD31 width (*μ*m)	20 ± 22	16 ± 7	50 ± 21^∗^°	18 ± 9^∞^	27 ± 13	3.562	0.009
*α*-SMA stroma (*n*)	7 ± 5	23 ± 17^∗^	26 ± 9^∗^	10 ± 4	33 ± 20^∗^^§^	16.097	<0.001
*α*-SMA vessel (*n*)	4 ± 2	5 ± 3	4 ± 1	5 ± 3	5 ± 2	1.151	0.338
*α*-SMA width (*μ*m)	17 ± 22	15 ± 11	17 ± 15	28 ± 20	18 ± 16	2.031	0.119
Laminin length	3.58 ± 0.81	3.66 ± 0.62	3.33 ± 1.211	3.80 ± 0.63	3.91 ± 0.28		0.523
Laminin intensity	2.56 ± 0.83	3.22 ± 0.50^∗^	2.67 ± 0.81	3.20 ± 0.63^∗^	2.75 ± 0.75^∗^		0.004

Values are presented as mean ± SD. *p* < 0.05 is considered significant. Intensity values were averaged based on a scale of 1-4: 1 = 400x; 2 = 200; 3 = 100x; and 4 = 40x (4=strong and 1=weak); laminin expression along basement membrane length based on a scale of 0-4: 0=none; 1=1/4 length, 2=1/2 length, 3=3/4 length, and 4=full length. ^∗^Significantly different from control; °significantly different STSG, ^∞^significantly different from FTSG, and ^§^significantly different from Matriderm®.

## Data Availability

Access to data is restricted due to patient privacy.
